# A *BRCA1*-mutation associated DNA methylation signature in blood cells predicts sporadic breast cancer incidence and survival

**DOI:** 10.1186/gm567

**Published:** 2014-06-27

**Authors:** Shahzia Anjum, Evangelia-Ourania Fourkala, Michal Zikan, Andrew Wong, Aleksandra Gentry-Maharaj, Allison Jones, Rebecca Hardy, David Cibula, Diana Kuh, Ian J Jacobs, Andrew E Teschendorff, Usha Menon, Martin Widschwendter

**Affiliations:** 1 Department of Women’s Cancer, UCL Elizabeth Garrett Anderson Institute for Women’s Health, University College London, 74 Huntley Street, London WC1E 6 AU, UK; 2 Gynecological Oncology Center, Department of Obstetrics and Gynecology, Charles University in Prague – First Faculty of Medicine and General University Hospital, Apolinarska 18, 128 00 Prague, Czech Republic; 3 MRC Unit for Lifelong Health and Ageing at UCL, 33 Bedford Place, London WC1B 5JU, UK; 4 Faculty of Medical and Human Sciences, The University of Manchester, 46 Grafton Street, Manchester M13 9NT, UK; 5 Statistical Genomics Group, Paul O’Gorman Building, UCL Cancer Institute, University College London, 72 Huntley Street, London WC1E 6BT, UK; 6 CAS-MPG Partner Institute for Computational Biology Chinese Academy of Sciences, Shanghai Institute for Biological Sciences, Shanghai 200031, China

## Abstract

**Background:**

*BRCA1* mutation carriers have an 85% risk of developing breast cancer but the risk of developing non-hereditary breast cancer is difficult to assess. Our objective is to test whether a DNA methylation (DNAme) signature derived from *BRCA1* mutation carriers is able to predict non-hereditary breast cancer.

**Methods:**

In a case/control setting (72 *BRCA1* mutation carriers and 72 *BRCA1/2* wild type controls) blood cell DNA samples were profiled on the Illumina 27 k methylation array. Using the Elastic Net classification algorithm, a *BRCA1*-mutation DNAme signature was derived and tested in two cohorts: (1) The NSHD (19 breast cancers developed within 12 years after sample donation and 77 controls) and (2) the UKCTOCS trial (119 oestrogen receptor positive breast cancers developed within 5 years after sample donation and 122 controls).

**Results:**

We found that our blood-based *BRCA1*-mutation DNAme signature applied to blood cell DNA from women in the NSHD resulted in a receiver operating characteristics (ROC) area under the curve (AUC) of 0.65 (95% CI 0.51 to 0.78, *P* = 0.02) which did not validate in buccal cells from the same individuals. Applying the signature in blood DNA from UKCTOCS volunteers resulted in AUC of 0.57 (95% CI 0.50 to 0.64; *P* = 0.03) and is independent of family history or any other known risk factors. Importantly the *BRCA1*-mutation DNAme signature was able to predict breast cancer mortality (AUC = 0.67; 95% CI 0.51 to 0.83; *P* = 0.02). We also found that the 1,074 CpGs which are hypermethylated in *BRCA1* mutation carriers are significantly enriched for stem cell polycomb group target genes (*P* <10^-20^).

**Conclusions:**

A DNAme signature derived from *BRCA1* carriers is able to predict breast cancer risk and death years in advance of diagnosis. Future studies may need to focus on DNAme profiles in epithelial cells in order to reach the AUC thresholds required of preventative measures or early detection strategies.

## Background

Breast cancer is the most common cancer in women, affecting at least 1 in 10 women in the western world. The potential to predict breast cancer and offer preventive measures is an effective intervention in women with an inherited predisposition to breast cancer due to mutations in *BRCA1/2* genes [1]. However, these account for less than 10% of breast cancers [2]. While extensive genome-wide association studies have identified a number of single nucleotide polymorphisms (SNPs) associated with breast cancer risk [3], epidemiological models that include risk associated SNPs yield a receiver-operating-characteristic (ROC) area under the curve (AUC) of only 62%, a modest 4% improvement over the AUC of epidemiological models [4].

Predicting the likelihood of breast cancer development is therefore still challenging not only because the sensitivity of current strategies is low [4] but also because 11% to 52% of screen-detected breast cancers may be an over diagnosis of cancers which would have never become clinically evident [5-7]. Hence a biomarker that could predict the risk of developing breast cancer particularly in those with a poor prognosis and which is also independent of familial predisposition is urgently needed.

It is known that epigenetic variation contributes to inter-individual variation in gene expression and thus may contribute to variation in cancer susceptibility [8-10]. DNAme is the most studied mechanism of epigenetic gene regulation and represents a biologically and chemically stable signal. Aberrant DNA methylation is also a hallmark of cancer [9,11], in particular increased promoter DNAme at stem cell differentiation genes (Polycomb-Repressive Complex 2 (PRC2) Group Target genes (PCGTs)) [12-19]. Initial evidence suggests that *BRCA1* is a key negative modulator of PRC2 and that loss of *BRCA1* inhibits stem cell differentiation and enhances an aggressive breast cancer phenotype by affecting PRC2 function [20]. Several proof of principle studies using a target gene approach or assessment of global DNA methylation analysing samples collected at the time of diagnosis provided the first evidence for the feasibility of breast cancer risk prediction using DNA methylation based markers [21-32]. It was also recently demonstrated that DNAme profiles in blood are able to predict cancer risk (on average 1.3 years in advance) within a group of women whose sisters had developed breast cancer [33].

Here we tested the hypothesis that women with an extremely high breast cancer risk (due to a *BRCA1* mutation) carry a specific methylation signature in peripheral blood cells, which is also able to predict sporadic breast cancer incidence and death. We also tested whether this signature is tissue-specific.

## Methods

Data from three different studies were used.

### BRCA1 study

**Figure 1:**
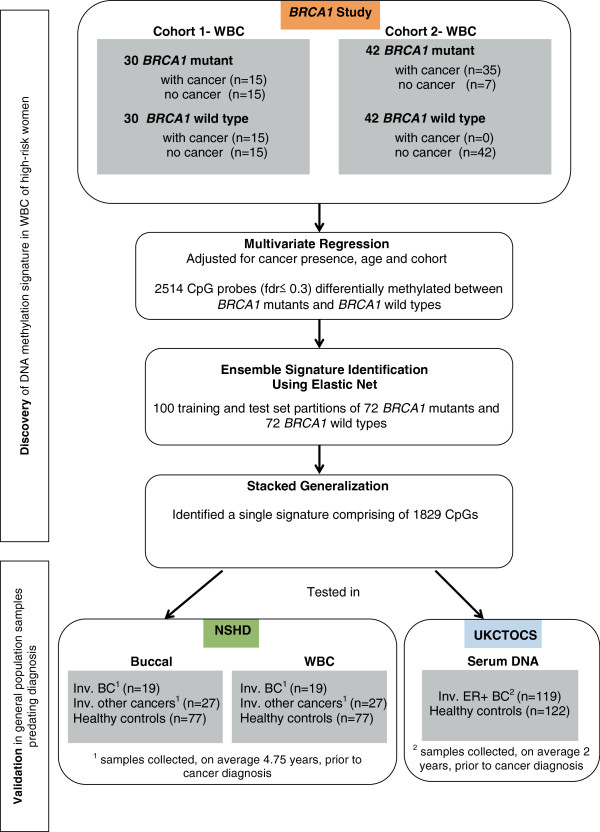
**Study design and identification/validation of the *BRCA1*-mutation DNAme risk signature.** AUC, receiver operating characteristics area under the curve; BC, breast cancer; FDR, false discovery rate; inv., invasive; WBC, white blood cells.

We analysed whole blood samples from two cohorts of *BRCA1* mutation carriers and controls without a *BRCA1* mutation (see Figure 1 and Additional file 1).

### MRC National Survey of Health and Development (NSHD)

We analysed both blood cells and buccal cells from a sample of women from the NSHD, a birth cohort study of men and women born in Britain in March 1946 [34-36]. A total of 152 (75 cancer cases and 77 controls) women were selected from those who provided both a peripheral blood and a buccal cell sample at the age of 53 years in 1999, who had not previously developed any cancer and who had complete information on epidemiological variables of interest and follow-up. We analysed >480,000 CpGs (using the Illumina 450 k array) in the 46 women who developed an invasive non-skin cancer (19 breast cancer, 5 reproductive tract and 22 other cancers; diagnosed 1 to 7 years after 53 years and an average of 4.75 years) and in the women (n = 77) who did not develop any cancer during the 12-year follow-up (for descriptive analysis see Additional file 2).

### United Kingdom Collaborative Trial of Ovarian Cancer Screening (UKCTOCS)

We analysed serum DNA samples (which largely represent white blood cell DNA in this cohort - see Additional files 3 and 4) from postmenopausal women who developed breast cancer (n = 119) or remained cancer-free during the follow-up period (n = 122, maximum of 12 year follow-up (2001 to 2013)).

### Ethics

All studies were approved by the relevant research ethics committee or institutional review board. Informed consent was obtained by all volunteers and conforms with the Declaration of Helsinki. The *BRCA1* study was approved by the ethics committee of the General University Hospital, Prague (No. 1199/07 S-IV). The NSHD epigenetics study was approved by the Central Manchester Research Ethics Committee (REC reference: 07/H1008/168). UKCTOCS was approved by the UK North West Multicentre Research Ethics Committees (North West MREC 00/8/34). Ethical approval for this nested case control study was obtained from the Joint UCL/UCLH Committees on the Ethics of Human Research (REC reference: 06/Q0505/102).

### DNA methylation analysis

The DNA from whole blood and tissues was extracted at UCL [36] and at Gen-Probe [37]. Methylation analysis was performed using the validated Illumina Infinium Human Methylation27 BeadChip [16] or the Illumina Infinium Human Methylation450 BeadChip for NSHD samples. The methylation status of a specific CpG site was calculated from the intensity of the methylated (M) and unmethylated (U) alleles, as the ratio of fluorescent signals β = Max(M,0)/(Max(M,0) + Max(U,0) + 100). On this scale, 0 < β < 1, with β values close to 1 (0) indicating 100% methylation (no methylation) (see Additional file 4).

### Data availability

Data from two of the studies in this manuscript have been deposited in the Gene Expression Omnibus repository under the accession numbers (GSE58119), (GSE57285), (GSE32396). The NSHD data are made available to researchers who submit data requests to mrclha.swiftinfo@ucl.ac.uk; see full policy documents at [38]. Managed access is in place for this 68-year-old study to ensure that use of the data is within the bounds of consent given previously by participants, and to safeguard any potential threat to anonymity since the participants are all born in the same week.

## Statistical analyses

### Differential methylation analysis

From the *BRCA1* study, differentially methylated CpGs, with false discovery rate (FDR) corrected *P* values, between *BRCA1* mutant carriers and *BRCA1* wild type samples were identified via a multivariate logistic regression that was adjusted for age, batch and the presence of cancer.

### Ensemble signature identification

The elastic net classification method was chosen for our study as it has been shown to be particularly effective when the number of predictors is far greater than the number of training points [39]. The elastic net method, as implemented in the glmnet R-package [40], identified a classifier comprising 1,829 CpGs with non-zero regression coefficients (see Additional file 4).

### Validation

To evaluate its predictive accuracy, the identified classifier was tested on two independent datasets: (1) NSHD, and (2) UKCTOCS. For each individual, risk scores, based on their methylation profiles, were estimated and correlated to their disease status. An AUC value was then obtained via Somers’ Dxy rank correlation [41] (see Additional file 4).

## Results

### DNA methylation signature in white blood cells (WBC) associated with *BRCA1* mutation status

**Figure 2:**
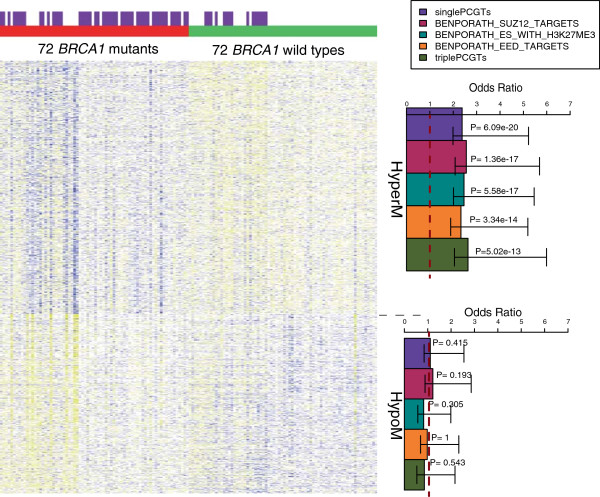
**CpGs (n = 1829), which are differentially methylated in WBCs between *BRCA1 *mutation carriers and wild type controls and which comprise the ‘ *BRCA1 *-mutation DNA methylation signature’.** Heatmap of normalised methylation values (blue = relative high methylation, yellow = relative low methylation) of CpGs comprising the *BRCA1*-mutation DNAme signature. The first colour bar at the top denotes the two main clusters where ‘red’ reflects the samples with a *BRCA1* mutation whereas ‘green’ reflects samples without a mutation in *BRCA1* or *BRCA2* gene. The distribution of cancer cases is given in the second colour bar indicating women who had developed a breast cancer in purple. Right panel shows the enrichment of the top components of the gene set enrichment analysis in the hyper- and hypomethylated subset of CpGs; PCGT; Polycomb repressor complex 2 Group Target. Dashed line separates hypermethylated from hypomethylated CpGs.

We analysed DNAme of 27,578 CpGs in WBC samples from a total of 72 women with a known *BRCA1* mutation and 72 women with no mutation in the *BRCA1* or *BRCA2* gene (Figure 1 and Additional file 1). The presence of a cancer has been shown to modulate the composition of WBCs and DNAme profiles in peripheral blood [42] and hence we used a mixture of women who did and who did not develop breast cancer in order to be able to adjust for this. Using a multivariate regression model that included age, cohort and cancer status as covariates we were able to rank CpGs according to the significance of the association between their DNAme profile and mutation status. On applying a relaxed threshold of FDR <0.3 we observed a total of 2,514 *BRCA1*-mutation associated CpGs, of which 1,422 (57%) were hypermethylated (hyperM) and 1,092 (43%) were hypomethylated (hypoM) in women who had a *BRCA1* mutation (Figure 1, Additional file 5), representing a highly significant skew towards hypermethylated CpGs (Binomial test *P* < 1e-10). To arrive at a specific DNAme signature, which would allow classification of independent samples, we used the elastic net (ELNET) framework (see Additional file 4), which resulted in a signature consisting of 1,829 CpGs (Figure 2, Additional file 6).

Given that PCGT methylation is a hallmark of almost all cancers and that a *BRCA1* defect in normal non-neoplastic cells is likely to silence PCGTs and compromise cell differentiation [20], we posited that our *BRCA1* DNAme signature may be able to predict sporadic breast cancer. Interestingly, Gene Set Enrichment Analysis (GSEA) [43,44] on the 1,074 hypermethylated (Additional file 7) and 755 hypomethylated (Additional file 8) CpGs of the *BRCA1-*mutation signature demonstrated the association of *BRCA1* mutation with promoter hypermethylation of PCGTs. Indeed, the top categories of genes, associated with the hypermethylated CpGs in *BRCA1* mutation carriers, were significantly (*P* <10^-10^) enriched for stem cell PCGTs irrespective of the definition used (Figure 2, Additional file 7). In contrast, none of the gene categories associated with those CpGs which are hypomethylated in *BRCA1* mutation carriers reached significance based on adjusted *P* values (Additional file 8). Even the GSEA on the 105 CpGs with a more stringent FDR (<=0.05) associated with *BRCA1* mutation in white blood cells demonstrated the enrichment of PCGTs (*P* < =0.02) (Additional file 9).

### *BRCA1*-mutation DNAme signature and breast cancer risk in peripheral blood cells in the NSHD

**Figure 3:**
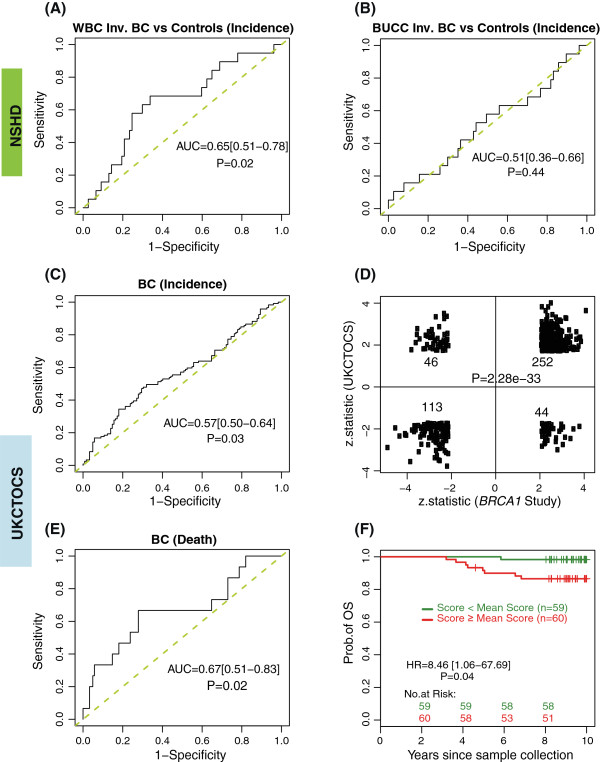
**Validation of the *BRCA1*-mutation DNAme signature in two independent prospective cohorts.** ROC curves and AUC statistics to predict future breast cancer (BC) incidence applying the *BRCA1*-mutation DNAme signature in white blood cells (WBCs) **(A)** and in buccal (BUCC) cells **(B)** of the NSHD cohort and in serum DNA of the UKCTOCS cohort **(C)**. Overlap of the top CpGs differently methylated in WBC between *BRCA1* mutant and wild type (*BRCA1* study) and the top CpGs differently methylated in serum DNA between women who have developed oestrogen receptor positive BCs and women who remained cancer-free **(D)**. ROC curve and AUC statistics to predict deadly BCs applying the *BRCA1*-mutation DNAme signature in serum DNA in the UKCTOCS cohort **(E)** and Kaplan Meier curve (and hazard ratio (HR)) of future breast cancer patients with a high and low *BRCA1*-mutation DNAme score in serum DNA **(F)**.

In order to test whether the *BRCA1*-mutation DNAme signature is able to identify women who will develop breast cancer we analysed one of the best available characterised longitudinal cohorts (Additional file 2). Applying the *BRCA1*-mutation DNAme signature (out of the 1,829 *BRCA1* CpGs, 1,722 were present on the 450 k Illumina methylation array), yielded a breast cancer risk AUC = 0.65 (0.51 to 0.78, *P* = 0.02) (Figure 3A). Interestingly, the *BRCA1* signature also significantly predicted the future development of invasive non-breast cancers (AUC = 0.62; 0.50 to 0.74; *P* = 0.04) (Additional file 10A).

Consistent with the view that DNAme is tissue-specific, our DNAme signature - derived from peripheral blood cells from women with known *BRCA1* status - was not able to predict invasive breast cancer (Figure 3B) or invasive non-breast cancer (Additional file 10B) in the buccal cell DNAme profiles obtained at the same time from the same women who provided blood DNA.

### *BRCA1-*mutation DNA methylation signature and breast cancer risk in serum DNA in the UKCTOCS cohort

Less than 10% of invasive breast cancers are due to a *BRCA1* mutation [45] and therefore it is unlikely that the predictive capacity of the *BRCA1*-mutation DNAme signature in the NSHD cohort was due to the correct identification of *BRCA1* mutation carriers. Nevertheless in order to further substantiate that the *BRCA1*-mutation DNAme signature identifies sporadic cancers, we performed a nested case–control study within the UKCTOCS cohort (a 202,638 postmenopausal women cohort, who based on their family history were not at an increased risk of ovarian or breast cancer - see Additional files 3 and 4). As *BRCA1*-associated cancers are far more likely (75%) to be oestrogen receptor (ER) negative [46], we solely focused our analysis on women who provided a blood sample between 0.42 and 4.18 years (average 2 years) before they developed an ER positive invasive breast cancer (n = 119) and matched (on age at blood donation and recruitment centre) them to 122 women who did not develop a breast cancer during the follow-up period (5.61 to 12 years, average follow-up 11.92 years). As there was no whole blood DNA samples available from the women in UKCTOCS, we used serum-free DNA as a source of material for this analysis. Since >95% of blood samples were only spun down 24 to 48 h after the blood draw, it was important for us to identify the likely source of DNA in the serum samples. Although we were not able to definitely identify the source, the evidence clearly pointed towards an enriched for WBC DNA (see Additional file 11). The *BRCA1*-mutation DNAme signature predicted the development of an ER positive breast cancer with an AUC = 0.57 (0.50 to 0.64; *P* = 0.03) (Figure 3C) independent of whether the sample was taken less or more than 2 years prior to diagnosis (see Additional file 12). Importantly, the *BRCA1-*mutation DNAme signature also substantially overlapped with an ER + breast cancer specific risk signature (Additional file 13), which we derived *de novo* in the UKCTOCS cohort (*P* <2 x 10^-33^, Figure 3D). Of note, in the breast cancer specific risk signature, we also observed enrichment of biological terms, all crucially involved in stem cell differentiation and biology (Additional file 14). Again, these stem cell gene categories were only enriched among CpGs hypermethylated in cases, but not among CpGs hypomethylated in cases (Additional file 15). This observation is particularly pertinent given that NIPP1, PRC2, MSX1 and NANOG all suppress differentiation through occupation and suppression of specific gene sets.

### *BRCA1*-mutation DNAme signature identifies women years in advance of fatal breast cancer diagnosis

In order to test whether the *BRCA1*-mutation DNAme signature is able to predict not only incidence but also breast cancer mortality we performed ROC statistics in the UKCTOCS set comparing women who died from breast cancer (n = 10) during the follow-up period with women who did not develop breast cancer (Figure 3E) and found an AUC = 0.67 (0.51 to 0.83; *P* = 0.02). In line with these findings women with a higher than average *BRCA1*-mutation DNAme signature score were 8.46 (95% CI 1.06 to 67.69) -fold more likely to die from breast cancer (*P* = 0.04) than those with lower than average scores (Figure 3F). Interestingly, apart from the number of nodes, none of the other clinico-pathological features or treatment modalities was associated with the *BRCA1*-mutation DNAme signature in these ER positive breast cancers (Additional file 16).

### *BRCA1*-mutation DNAme signature and association with epidemiological and hormonal risk markers

Next, we were interested whether our DNAme signature could be explained by any of the breast cancer risk factors we had available for the UKCTOCS cohort. Interestingly, neither any of the epidemiological breast cancer risk factors nor any of the hormones (Tables 1, 2 and 3) we have analysed in the same serum samples was associated with our *BRCA1*-mutation DNAme signature. Interestingly, when we analysed women with and without a family history [47] separately, both BC incidence and death was predicted by our *BRCA1*-DNAme signature only in the group without family history (Additional file 17), but not in the (obviously very small) group of women with any family history (Additional file 18).

**Table 1:** Characteristics of the samples used from the UK Collaborative Trial of Ovarian Cancer Screening (UKCTOCS)

**Factors**		**Methylation signature**		***P *** **value**
		**Positive**	**Negative**	
OCP use in the past	Yes	72	60	0.604
	No	55	54	
Pregnancies <6 months	Yes	32	35	0.387
	No	95	78	
Pregnancies >6 months	Yes	109	102	0.439
	No	18	12	
Mother breast cancer	Yes	14	11	0.833
	No	113	103	
Grandmother(s) breast cancer	Yes	2	7	0.0887
	No	125	107	
Sister(s) breast cancer	Yes	5	11	0.118
	No	122	103	
Aunt(s) breast cancer	Yes	12	6	0.233
	No	115	108	
Any family member breast cancer	Yes	29	32	0.376
	No	98	82	
Alcohol units per week	Yes	78	68	1
	No	26	23	
Smoker	Yes	41	37	0.89
	No	84	72	

The samples were categorised according to their individual risk scores. These risk scores are the product of the methylation profile with the regression coefficients of the signature. The statistical significance was assessed by a two-sided, Fisher’s exact test. The missing values were excluded from the analysis.

**Table 2:** Additional characteristics of the samples used from the UK Collaborative Trial of Ovarian Cancer Screening (UKCTOCS)

	**Breast cancer (#Samples)**	**Methylation signature**		***P *** **value**
		**Positive (SD)**	**Negative (SD)**	
Mean BMI (kg/m^2^)	Yes (119)	27.39 (5.3)	27.27 (5.0)	0.9
	No (121)	26.63 (5.17)	26.57 (4.64)	0.95
Mean age at menarche (years)	Yes (117)	12.89 (1.48)	12.65 (1.61)	0.4
	No (122)	12.97 (1.76)	13.24 (1.77)	0.39
Mean age at menopause (years)	Yes (119)	49.58 (5.78)	48.37 (7.65)	0.34
	No (122)	47.57 (7.99)	48.74 (5.79)	0.36

The samples were categorised according to their individual risk scores. These risk scores are the product of the methylation profile with the regression coefficients of the signature. The statistical significance was assessed by a t-test. The missing values were excluded from the analysis.

**Table 3:** Characteristics of the samples used from the UK Collaborative Trial of Ovarian Cancer Screening (UKCTOCS)

**Hormones**	**Breast cancer (#Samples)**	**Methylation signature**		***P *** **value**
		**Positive (SD)**	**Negative (SD)**	
Mean oestradiol, pg/mL	Yes (65)	20.51 (16.74)	18.67 (10.09)	0.59
	No (115)	17.36 (8.32)	19.24 (8.11)	0.22
Mean free oestradiol, pmol/L	Yes (65)	0.98 (0.58)	0.99 (0.55)	0.95
	No (114)	0.84 (0.43)	1.02 (0.59)	0.05
Mean oestrone, pg/mL	Yes (67)	126.7 (156.59)	97.22 (58.10)	0.31
	No (117)	112.7 (91.86)	97.55 (92.41)	0.38
Mean androstendione, nmol/L	Yes (64)	3.35 (1.66)	3.96 (2.00)	0.19
	No (118)	3.4 (2.10)	3.13 (1.41)	0.41
Mean testosterone, nmol/L	Yes (65)	0.30 (0.17)	0.35 (0.20)	0.3
	No (115)	0.28 (0.17)	0.31 (0.19)	0.42
Mean free testosterone, ng/dl	Yes (65)	0.12 (0.08)	0.14 (0.10)	0.35
	No (115)	0.11 (0.07)	0.13 (0.09)	0.17
Mean SHBG, nmol/L	Yes (66)	57.54 (37.71)	49.79 (19.86)	0.24
	No (116)	61.26 (26.46)	54.49 (24.36)	0.16
Mean progesterone, ng/mL	Yes (66)	0.63 (2.13)	0.28 (0.21)	0.34
	No (114)	0.27 (0.17)	0.28 (0.16)	0.84
Mean DHEAS, ug/dl	Yes (66)	109.6 (57.54)	92.52 (56.75)	0.23
	No (116)	115.8 (65.49)	107.1 (57.92)	0.45
Mean ER alpha, pg/mL	Yes (67)	89.67 (87.33)	81.23 (56.48)	0.64
	No (120)	69.54 (62.56)	75.29 (61.84)	0.61
Mean ER beta, pg/mL	Yes (67)	87.06 (122.37)	64.01 (75.85)	0.35
	No (120)	56.74 (62.83)	60.95 (72.14)	0.73
Mean AR, ng/mL	Yes (67)	2.5 (0.95)	2.4 (0.91)	0.78
	No (119)	2.28 (1.11)	2.45 (0.96)	0.37

The samples were categorised according to their individual risk scores. These risk scores are the product of the methylation profile with the regression coefficients of the signature. The statistical significance was assessed by a t-test. The missing values were excluded from the analysis.

## Discussion

Here we have provided several novel lines of evidence indicating that DNAme profiles obtained in cells from women with a *BRCA1* mutation have the potential to indicate future breast cancer development (and death) many years in advance of diagnosis. Our findings also show that genes encoding developmental transcription factors integral for stem cell differentiation and biology are hypermethylated in women predisposed to breast cancer.

Our data suggest that the *BRCA1*-associated DNAme signature is a risk predicting signature rather than an early detection signature, because: (1) the DNAme signature was derived from WBCs in women with a known *BRCA1* status and was adjusted for cancer status (analysis included *BRCA1* carriers without cancer at the time of sample draw); (2) the time from sample draw to diagnosis had no dramatic impact on the strength of association between DNAme and potential for breast cancer development; (3) the signature was validated in two independent cohorts; (4) we observed a very strong overlap of CpGs associated with *BRCA1* mutation (*BRCA1* study) and CpGs indicating future breast cancer risk (UKCTOCS); and finally (5) the signature was also associated with invasive non-breast cancers.

The observation that the top ranked hypermethylated *BRCA1*-mutation associated CpGs are highly enriched for PCGTs which we and others have previously shown to be an epigenetic hallmark of cancer tissue [12-18] and which are among the earliest, if not the earliest, molecular changes in human carcinogenesis [18] was an exciting finding because it fully supports recent data demonstrating that a *BRCA1* defect leads to retargeting of the PRC2 and reduces cell differentiation.

Two key issues remain unclear. First, which factors lead to a *BRCA1*-mutation DNAme pattern in the absence of a *BRCA1* mutation? It is likely that a combination of risk factors or factors which we have not captured (for example, early life events, transgenerational inheritance, and so on) contribute to epigenetic modifications which are in common to those associated with *BRCA1* mutation. Second, is the *BRCA1*-mutation DNAme signature in WBCs functionally relevant or just simply an indicator of breast cancer risk? The fact that the signature is indicative of breast cancer mortality would support the view that subtle epigenetic mis-programming of immune cells may lead to general immune defects which in turn supports the development and proliferation of cancers. However, all these suggestions are highly speculative and need validation in further independent cohorts using well-defined subsets of blood cells or epithelial cells.

There are limitations to this study. First, we analysed whole blood DNA or serum DNA representing whole blood DNA and not a specific subset of peripheral blood cells. Second, although we found some good preliminary evidence that DNAme profiles in buccal cells are better at predicting future breast cancer risk (data not shown), we did not analyse buccal cells from *BRCA1* mutation carriers, nor did we have access to independent prospective buccal cell data. Third, we used the 27 k array, instead of the 450 k array, to generate the *BRCA1*-mutation DNAme signature.

In summary, our data highlight DNAme analysis as a promising tool to predict future breast cancer development. Future epigenome-wide studies should focus on using epithelial cells like buccal - or epithelial cells from the uterine cervix which are hormone sensitive and more likely to capture an ‘epigenetic record’ of breast cancer risk factors. Such studies are more likely to provide the level of specificity and sensitivity which is required for a clinically useful risk prediction tool.

## Conclusions

In summary, our DNAme signature derived from blood cells from *BRCA1* carriers is able to predict breast cancer risk and death years in advance of diagnosis albeit with a modest AUC. Our data further support the notion that DNAme modification at stem-cell differentiation genes, even in unrelated tissues, is an early event associated with carcinogenesis.

## Abbreviations

AUC: Area under the curve; DNAme: DNA methylation; FDR: False discovery rate; GSEA: Gene Set Enrichment Analysis; hyperM: Hypermethylated; hypoM: Hypomethylated; ROC: Receiver operating characteristics.

## Competing interests

IJ had a consultancy arrangement with Becton Dickinson in the field of tumour markers and ovarian cancer. Both IJ and UM have a financial interest through UCL Business and Abcodia Ltd. in the third party exploitation of clinical trials biobanks which have been developed through the research at UCL. The remaining authors declare that they have no competing interests.

## Authors’ contributions

MW and SA had full access to all of the data in the study and take responsibility for the integrity of the data and the accuracy of the data analysis. Study concept and design: MW. Acquisition of data: EOF, MZ, AW, AGM, RH, DC, DK, UM, IJ. Analysis and interpretation of data: SA, AJ, AET, MW. Drafting of the manuscript: MW. Critical revision of the manuscript for important intellectual content: AET, SA, EOF, MZ, AW, AGM, AJ, RH, DC, DK, UM, IJ. Statistical analysis: SA. Obtained funding: MW, IJ, UM, DK. Administrative, technical or material support: SA. Study supervision: MW, AET, DK, IJ, UM. All authors read and approved the final manuscript.

## Supplementary Material

Additional file 1 Description of the BRCA1 Study (White Blood Cell DNA samples from the Charles University in Prague). Click here for file

Additional file 2 Characteristics of the sample from the MRC National Survey of Health and Development study (NSHD). Click here for file

Additional file 3 **Characteristics of the samples used from the UK Collaborative Trial of Ovarian Cancer Screening (UKCTOCS).** Statistical significance was tested using a two-sided, Fisher’s exact test. The missing values were not included in the analysis. Click here for file

Additional file 4 Supplementary Information Document with further details of Materials and Methods. Click here for file

Additional file 5 **Top ranked 2514 CpGs in white blood cells associated with *BRCA1* mutation.** These differentially methylated CpGs were identified based on their β-value methylation profiles. This was done via a multivariate logistic regression that was adjusted for age, batch and the presence of cancer. To correct for multiple hypothesis testing, the false discovery rates (FDR) were estimated using the q-value analytical procedure available through the R qvalue Bioconductor package. Click here for file

Additional file 6 ***BRCA1*-mutation associated DNA methylation signature consisting 1829 CpGs.** The signature was derived by combining, via stacked generalisation, 100 cross-validated classifiers using ElasticNet with an alpha = 0.1. Click here for file

Additional file 7 **Gene Set Enrichment Analysis on the top 1074 hypermethylated CpGs of the *BRCA1* DNA methylation signature.** Click here for file

Additional file 8 **Gene Set Enrichment Analysis on the top 755 hypomethylated CpGs of the *BRCA1* DNA methylation signature.** Click here for file

Additional file 9 **Gene Set Enrichment Analysis on the 105 differentially methylated CpGs (FDR < =0.05) in white blood cells associated with *BRCA1* mutation.** Click here for file

Additional file 10 ROC curve for the identified signature in invasive non-breast cancer samples of the NSHD dataset. Click here for file

Additional file 11 **Average number of differentially methylated CpGs between tissue types.** UKOPS WB = whole blood from postmenopausal healthy women; T1D WB = whole blood from pre and postmenopausal women with type-1 diabetes. Click here for file

Additional file 12 ROC curve of the identified signature on the UKCTOCS dataset, separated around the diagnosis time of less than/greater than 2 years. Click here for file

Additional file 13 Top ranked 5482 CpGs associated with future breast cancer risk from serum DNA in the UKCTOCS set. Click here for file

Additional file 14 Gene Set Enrichment Analysis on the top 3395 hypermethylated CpGs in the UKCTOCS cohort using a multivariate linear regression framework. Click here for file

Additional file 15 Gene Set Enrichment Analysis on the top 2087 hypomethylated CpGs in the UKCTOCS cohort using a multivariate linear regression framework. Click here for file

Additional file 16 **Characteristics of the samples used from the UK Collaborative Trial of Ovarian Cancer Screening (UKCTOCS).** The samples were categorised according to their individual risk scores. These risk scores are the product of the methylation profile with the regression coefficients of the signature. The statistical significance was assessed by a two-sided, Fisher’s exact test. The missing values were not included in the analysis. Click here for file

Additional file 17 ROC curve of the identified signature on the UKCTOCS dataset samples, without a family history of breast cancer, separated into breast cancer incidence and breast cancer mortality. Click here for file

Additional file 18 ROC curve of the identified signature on the UKCTOCS dataset samples, with a family history of breast cancer, separated into breast cancer incidence and breast cancer mortality. Click here for file
