# Supplementary material for: A BRCA1-mutation associated DNA methylation signature in blood cells predicts sporadic breast cancer incidence and survival
Source: Genome Med. 2014 Jun 27;6(6):47. doi: 10.1186/gm567 (PMC4110671; doi:10.1186/gm567)
Supplement: Additional file 4 — Supplementary Information Document with further details of Materials and Methods. [file gm567-S4.pdf]

SUPPLEMENTARY INFORMATION

for manuscript

A *BRCA1*-mutation associated DNA methylation signature in blood cells predicts sporadic breast cancer incidence and survival

Anjum S, Fourkala EO, Zikan M, Wong A, Gentry-Maharaj A, Jones A, Hardy R, Cibula D, Kuh D, Jacobs IJ, Teschendorff AE, Menon U and Widschwendter M

STUDY POPULATION

(A) UKCTOCS serum DNA samples

The serum samples in this study were drawn from the UK Collaborative Trial of Ovarian Cancer Screening (UKCTOCS)[1], which is one of the largest prospectively randomised clinical trials and recruited more than 200,000 women. The trial aims to assess the impact of screening on mortality from ovarian cancer and to comprehensively evaluate its physical and psychological morbidity, compliance rates and financial costs, as well as performance characteristics of its various screening strategies. The trial was set up at 13 centres in England, Wales and Northern Ireland.

All women in the trial were postmenopausal aged 50-74 and recruited between 2001 and 2005 through random invitation from age/sex registers of the above local health authorities. The study population was massively depleted of women with familial breast and ovarian cancer risk.

(B) BRCA1 study- white blood cell (WBC) samples from *BRCA1* mutation carriers and *BRCA1* wild type controls

Whole blood samples were drawn from 15 healthy *BRCA1* mutation carriers (mean age 57.13) and 15 age-matched healthy females without a *BRCA1* mutation (mean age 56.8, Wilcoxon test p-value=0.66). An additional independent set of blood samples were drawn from 7 healthy *BRCA1* mutation carriers (mean age 30.43) and 42 healthy females without a *BRCA1* mutation (mean age 43.57, Wilcoxon test p-value=0.016). All samples were collected between 2001 and 2008. The samples were drawn from women attending the General Faculty Hospital in Prague. All women gave their written informed consent. This study has been approved by the ethics committee of the General University Hospital, Prague.

Blood samples were collected by the study nurses into vacuette tubes (7mL in K2 EDTA tubes) and were frozen within 3 hours of collection.

BRCA1 mutation testing in DNA from whole blood

Both *BRCA1* mutation carriers and controls (from the *BRCA1* Study) were tested for the presence of germ-line *BRCA1* mutation, including large genomic rearrangements.

Protein truncation test (PTT) was used as a prescreening method for exon 11 and direct sequencing was used as a detection method for exons 2-10 and 12-24 (exons 1a and 1b are non-coding exons and are not analyzed in standard protocols). All the mutations detected were confirmed by direct sequencing using at least two different primers on original and re-sampled DNA. Multiplex ligation-dependent probe amplification (MLPA) was used to detect large genomic rearrangement. Long-range PCR, isolation of certain alleles and direct sequencing were used to identify break-point of those rearrangements[2].

(C) NSHD white blood cell (WBC) samples and buccal samples

The National Survey of Health and Development study, with data on over 5000 people born in a single week of March 1946, is the longest-running birth-cohort study in the world. The Medical Research Council (MRC) currently runs this study.

For the purposes of a separate ongoing study, on breast cancer risk, 800 buccal DNA samples (> 50 ng/µl) were taken from postmenopausal women at age 53. Out of these 800 women, blood samples (DNA conc > 30 ng/µl), also at the same time point, were available for 200 women.

Information on certain variables such as digitized mammogram, age at puberty, parity and menopause status, were deemed essential for the proposed breast cancer risk study. Hence, only those women (n=798) with non-missing data for all the essential variables were selected.

On the 200 women, with corresponding blood samples, an additional criterion of either cancer registration after 1999 or healthy was applied. This narrowed the total blood DNA samples to 77 women with cancer and 212 without cancer. Out of the 212 healthy women, 77 women were chosen such that they were representative of the percent breast density (0 – 77.78%) observed in all 212 healthy post-menopausal women.
Both the blood and buccal samples were stored at -20°C until required for processing. At which point the samples were defrosted at room temperature for approximately 1 hour. The sample plates were placed on a plate mixer for 4 minutes followed by a 1-minute spin down.

DNA extraction

The DNA from whole blood and tissues[3] was extracted using a chloroform based extraction method from 400µL of blood and Qiagen DNeasy Blood & Tissue Kit (69504), respectively. The DNA from 500µL serum (UKCTOCS) was extracted at Gen-Probe

(www.gen-probe.com), using Qiagen QiAamp Blood Mini Kit (51106). The overall average amount of DNA in the samples was 100-720ng and average 234ng. Most of the DNA in our serum samples is likely to be blood cell DNA.

BS modification
All DNA samples were bisulphite modified using the EZ DNA Methylation Kit D5008 (Zymo Research, Orange, CA, USA) according to the manufacturer's instructions.

DNA methylation profiling
Methylation analyses for the data were performed using the validated Illumina Infinium Human Methylation27 BeadChip for the UKCTOCS and the *BRCA1* Study. For the NSHD, Illumina **HumanMethylation450 BeadChip** was used. In all instances, for the assay, bisulphite (BS) converted DNA is amplified, fragmented and hybridised to the BeadChip arrays (each chip accommodates 12 samples as designated by Sentrix positions A-L). A single base extension is then performed using labelled DNP- and biotin labelled dNTPs. The arrays were imaged using a BeadArray Reader. Image processing and intensity data extraction were performed according to Illumina's instructions. Each interrogated locus is represented by specific oligomers linked to two bead types: one representing the sequence for methylated DNA (M) and the other for unmethylated DNA (U). For each specific CpG site, the methylation status is calculated from the intensity of the M and U alleles, as the ratio of the fluorescent signals β = Max(M,0) / [Max(M,0)+Max(U,0)+100]. Hence, DNA methylation β-values are continuous variables between 0 (absent methylation) and 1 (completely methylated) representing the ratio of the methylated allele to the combined locus intensity.

Quality control (QC) and Data Normalization

The quality of any given sample run can be assessed using the built-in controls. In addition, every methylation value measured on the array is accompanied by a detection p-value. Threshold p-value above 0.01 have been previously reported as being unreliable[4] and therefore have been filtered out. Missing β-value data were imputed using the k-nearest neighbours procedure[5].

Following QC, the data was normalized using the subset-quantile within array normalization (SWAN) method[6].

Ensemble Signature Identification
Identification of a methylation signature that is predictive of sporadic breast cancer is essentially a classification problem. This problem can be formulated as follows: Given a training data set, $\{(x_i, y_i)\}_{i=1}^{n}$, over n samples, where $x_i = (x_{i1}, x_{i2}, \ldots, x_{ip})$ is the input vector over p predictors and $y_i \in \{0,1\}$ is the binary outcome label; the aim is to learn a classification rule $f: R^n \rightarrow \{0,1\}$, that is capable of assigning an outcome label to a new and independent subject.
For the methylation data, the x_i represent the β-methylation profiles for the ith sample over p CpGs. The outcome label y_i represents the *BRCA1* mutation status where y_i=1 if the sample is a *BRCA1* mutant and y_i=0 otherwise.
This classification problem can be solved by regression methods such as lasso regression[7], SVM[8]and elastic net[9]. The elastic net classification method was chosen for our study as it has been shown to be particularly effective when the number of predictors is far greater than the number of training points[10].

The elastic net is a regularization technique that combines the L1-norm and L2-norm of

lasso and ridge regression. The estimates for this method are calculated by minimizing the following equation

$$\hat{\beta} = argmin_{\beta} \; \| y - X\beta \|^{2} + \lambda \left(\alpha \| \beta \|_{1} + (1-\alpha) \| \beta \|^{2}\right)$$

Where $\lambda > 0$ and $0 \leq \alpha \leq 1$ are the model parameters that control the sparsity of the solution. Setting $\alpha = 0$, leads the elastic net estimate back to the ridge regression estimate. The ridge (quadratic) part of the penalty stabilizes the L1 regularization path and removes the upper bound on the number of variables selected.

The elastic net method, as implemented in the glmnet R-package with a cyclical coordinate descent algorithm[9], was applied, with a penalization value of λ_1=0.1, to the list of 2514 differentially methylated CpGs identified in the *BRCA1* study. The penalization parameter value was selected such that between 100-200 CpGs were selected in the optimal classifier.

In our study, in order to arrive at a set of robust classifiers using the elastic net, the following steps were taken:

1. The *BRCA1* data was standardized, across the samples to mean zero, and standard deviation equal to one.
2. A random selection of 80% of the cases and controls was chosen as the training set. While the remaining 20% of the cases and controls formed the test set.
3. In the training set, a linear regression model of the β-methylation profiles against age, cancer presence and cohort, was fitted on each of the 2514 differentially methylated CpGs.
4. The residuals from the linear regression model, from step 3, were input to the elastic net classification algorithm, as implemented in the glmnet R-package[9].
5. For each choice of parameter **λ,** predicted risk values were estimated for each independent test sample, t, with standardized methylation profile β_t using the equation below:

   $$R_{t} = \sum_{c=1}^{N_{cpg}} \hat{\eta}_{c} \beta_{t,c}$$

   Where, N_{cpg} is the total number of CpGs and $\hat{\eta}_c$ is the regression coefficient estimated via the elastic net for CpG, c. Here, the summation can be calculated over all the CpGs as those with regression coefficient $\hat{\eta}_c = 0$ will not contribute to the overall estimate. The estimated risk scores for the test set are then correlated to their *BRCA1* mutation status to obtain an AUC value. For the AUC, the closely related Somers' Dxy rank correlation[11] is calculated. The parameter **λ,** with the best AUC performance is recorded along with the corresponding optimal classifier.
6. The above steps from 2-5, were repeated 100 times and for each run the optimal classifier and AUC were noted.

Together the set of 100 optimal classifiers, obtained by the above procedure, formed our ensemble signature. The elastic net approach encourages a strong grouping effect and hence, the signatures identified will also be strongly correlated[10]. This provides an

opportunity to combine the ensemble signature to a single, more easily interpretable, signature.

Stacked Generalization
Stacked generalization is a flexible method for combining multiple classifiers. The outputs of each of the 100 classifiers, in the ensemble signature, are viewed as data points in the feature space upon which a combiner function can be trained[12]. Although, stacked generalization has been previously shown to overfit[13], leading to poor overall performance; this limitation can be overcome via regularization[12]. Under regularization, the predictive accuracy is improved by a reduction in the variance of the error at the cost of slightly increasing the bias[14].

To apply the method of stacked generalization to our ensemble signature, the predictions of each of the 100 classifiers, on the *BRCA1* data, were aggregated and combined with the known labels to form a meta-data training set. This meta-data training set is then input into the elastic net, which is the chosen combiner function. Once again, for the elastic net, the glmnet R-package was used[9]. The optimal parameters for the elastic net were selected via 10-fold cross-validation over a coarse grid for $\alpha = \{0,0.25,0.5,0.75,1\}$ and a finer grid of fraction= 0 to 0.4 in increments of 0.02 for the parameter λ. This procedure selected 23 out of the 100 classifiers in the ensemble signature, which were then averaged across the CpGs to arrive at the final single classifier. The final single classifier comprised of 1829 CpGs with non-zero regression coefficients.

Validation
To evaluate its predictive accuracy, the single classifier was tested on two independent data sets- 1) MRC set, and 2) UKCTOCS set.
For each independent test sample, t, once again the risk score was calculated as

$$R_{\mathrm{t}} = \sum_{c=1}^{N_{\mathrm{cpg}}} \hat{\eta}_{\mathrm{c}} \beta_{\mathrm{t,c}}$$

Where, N_{cpg} is the total number of CpGs. $\beta_{\mathrm{t,c}}$ is the standardized β-methylation vector for test sample t and CpG c. And $\hat{\eta}_{\mathrm{c}}$ is the estimated, non-zero regression coefficient for CpG, c, in the classifier. The estimated risk scores are then correlated to their disease status and an AUC value is obtained via Somers' Dxy rank correlation[15]. To evaluate the statistical significance of the observed AUC, the validation procedure was repeated 100 times with randomly permutated phenotype labels. By counting the number of times the permutation testing yielded a better AUC than the one observed, a measure of its probability, to have arisen by chance, was obtained. The ROC curves were plotted with the help of the ROCR R-package[16].

Gene Set Enrichment Analysis
Gene set enrichment analysis (GSEA) is a test to assess if a gene set is over-represented in a candidate list. A gene set is a pre-established, category of genes grouped together by a common feature such as a molecular pathways or cellular component[17,18]. A total of 8567 gene sets, categorised by common gene ontology, molecular pathways, chromosomal locations, or targets of regulatory motifs and miRNAs, were derived from the Molecular Signatures Database (MSigDB)[19,20]. Two additional gene sets, included in the analysis, were of Polycomb group targets (PCGTs), defined either by single occupancy of SUZ12, or EED, or H3K27me3 in human

embryonic stem cells (hESCs); or triple occupancy of all three[21].

Since not all members of the gene sets were on the Illumina HumanMethylation27 array, we focused on the 8227 subset of gene sets that had over 60% representation. The GSEA analysis, for each gene set, was done by generating a two-by-two table comparing the number of genes in the candidate list that also belong to the gene set with those that are not members. The significance of the over-representation was then assessed by a Fisher's exact test and adjusted for multiplicity by the Benjamini-Hochberg procedure.

Survival analysis

The survival analysis of the samples in the UKCTOCS study was modelled using the Cox proportional hazards model[22]. Here, the hazard function measures the importance of the calculated risk scores on the survival times since sample collection. Under the Cox proportional hazards model the hazard function for each individual is given by the equation below:

$$h_i(t) = h_0(t) * exp\{\beta X_i\}$$

Where, $h_0(t)$ is the baseline hazard for individual, i, at the survival time t. The risk score covariate, X, for each individual, i, enters the model linearly with a coefficient β. To validate the assumption of proportional hazards, the R cox.zph() function from the survival() library, was used (P=0.401) where a statistically significant pvalue implies violation of the proportional hazard assumption.

The Cox proportional hazards regression model was then fitted to the survival times and cancer status with the risk scores as a predictor. The risk scores were divided in to two groups separated around the mean and the modelling was done using the R coxph() function. The results of this survival analysis for the two risk groups are shown in Figure 3(F) using the Kaplan-Meir curve plot. The Kaplan-Meier plot is a graphical representation of the survival function as a sequence of step-wise estimates[23]. The cumulative probability of survival is shown on the y-axis. While, the x-axis represents the serial time and the lengths of the horizontal lines are indicative of the survival durations. A short vertical line marks the event of interest, which is death in this case.

REFERENCES

1. Menon U, Gentry-Maharaj A, Ryan A, et al. Recruitment to multicentre trials--lessons from UKCTOCS: descriptive study. *BMJ*. 2008;337:a2079.
2. Pohlreich P, Zikan M, Stribrna J, et al. High proportion of recurrent germline mutations in the BRCA1 gene in breast and ovarian cancer patients from the Prague area. *Breast cancer research : BCR*. 2005;7(5):R728-736.
3. Rousseau K, Vinall LE, Butterworth SL, et al. MUC7 haplotype analysis: results from a longitudinal birth cohort support protective effect of the MUC7*5 allele on respiratory function. *Annals of human genetics*. Jul 2006;70(Pt 4):417-427.
4. Sandoval J, Heyn H, Moran S, et al. Validation of a DNA methylation microarray for 450,000 CpG sites in the human genome. *Epigenetics*. 2011;6(6):692-702.
5. Leek JT, Storey JD. A general framework for multiple testing dependence. *Proc Natl Acad Sci U S A*. Dec 2 2008;105(48):18718-18723.
6. Maksimovic J, Gordon L, Oshlack A. SWAN: Subset-quantile within array normalization for illumina infinium HumanMethylation450 BeadChips. *Genome biology*. 2012;13(6):R44.
7. Tibshirani R. Regression shrinkage and selection via the Lasso. *J Roy Stat Soc B Met*. 1996;58(1):267-288.
8. Guyon I, Weston J, Barnhill S, Vapnik V. Gene selection for cancer classification using support vector machines. *Machine learning*. 2002;46(1-3):389-422.
9. Friedman JH, Hastie T, Tibshirani R. Regularization Paths for Generalized Linear Models via

Cooordinate Descent. *Journal of Statistical Software*. 2010;33(1).

10. Zou H, Hastie T. Regularization and variable selection via the elastic net. *Journal of the Royal Statistical Society* 2005;B.67:301-320.

11. Newson R. Confidence intervals for rank statistics: Percentile slopes, differences, and ratios. *Stata Journal*. 2006;6(4):497-520.

12. Reid SG, G. Regularized linear models in stacked generalization. Paper presented at: Proceedings of the 8th international workshop on multiple classifier systems 2009; Berlin, Heidelberg.

13. Ting KMW, I.H. Issues in stacked generalization. *Journal of Artificial Intelligence research*. 1999;10:271-289.

14. Hastie TT, R;Friedman,J. *The Elements of Statistical Learning*: Springer,Heidelberg; 2003.

15. R. N. Confidence intervals for rank statistics. *Stata Journal*. 2006;6:309-334.

16. Sing T, Sander O, Beerenwinkel N, Lengauer T. ROCR: visualizing classifier performance in R. *Bioinformatics*. 2005;21(20):3940-3941.

17. Mootha VK, Lindgren CM, Eriksson KF, et al. PGC-1alpha-responsive genes involved in oxidative phosphorylation are coordinately downregulated in human diabetes. *Nat Genet*. Jul 2003;34(3):267-273.

18. Subramanian A, Tamayo P, Mootha VK, et al. Gene set enrichment analysis: a knowledge-based approach for interpreting genome-wide expression profiles. *Proc Natl Acad Sci U S A*. Oct 25 2005;102(43):15545-15550.

19. Mootha VL, C;et.al. PGC-1α-responsive genes involved in oxidative phosphorylation are coordinately downregulated in human diabetes. *Nature Genetics*. 2003;34:267-273.

20. Subramanian AT, P;et.al. Gene set enrichment analysis: A knowledge-based approach for interpreting genome-wide expression profiles. *Proceedings of the National Academy of Sciences of the United States of America*. 2005;102(43):15545-15550.

21. Lee TI, Jenner RG, Boyer LA, et al. Control of developmental regulators by Polycomb in human embryonic stem cells. *Cell*. Apr 21 2006;125(2):301-313.

22. Cox DR, Oakes D. *Analysis of survival data*. London: Chapman and Hall; 1984.

23. Rich JT, Neely JG, Paniello RC, Voelker CC, Nussenbaum B, Wang EW. A practical guide to understanding Kaplan-Meier curves. *Otolaryngology--head and neck surgery : official journal of American Academy of Otolaryngology-Head and Neck Surgery*. Sep 2010;143(3):331-336.
