# Supplementary figures and images for: A BRCA1-mutation associated DNA methylation signature in blood cells predicts sporadic breast cancer incidence and survival
Source: Genome Med. 2014 Jun 27;6(6):47. doi: 10.1186/gm567 (PMC4110671; doi:10.1186/gm567)

(A)

WBC Inv. Non BC vs Controls (Incidence)

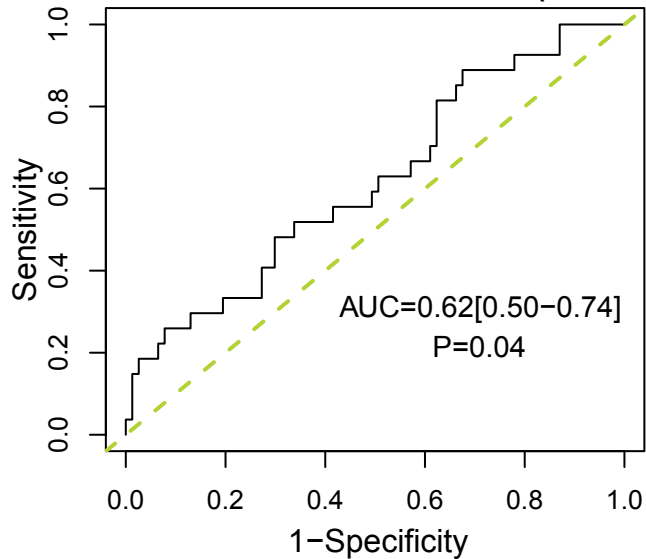

(B)

BUCC Inv. Non BC vs Controls (Incidence)

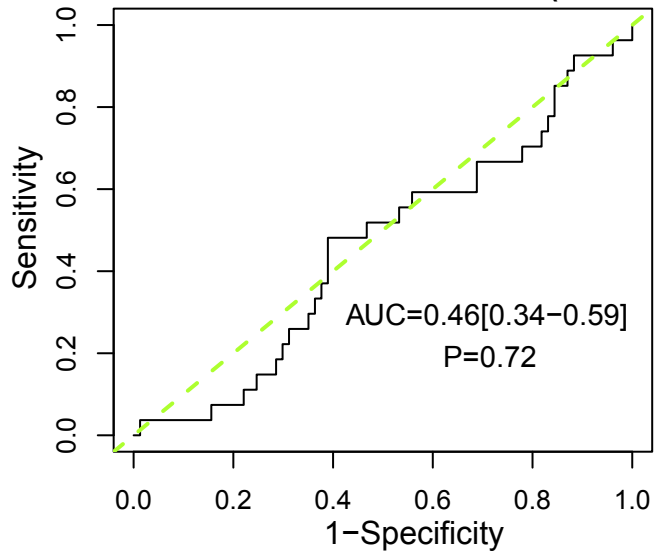

Supplement: Additional file 10 — ROC curve for the identified signature in invasive non-breast cancer samples of the NSHD dataset. [file gm567-S10.pdf]

(A)

BC Diagnosis (upto 2 years)

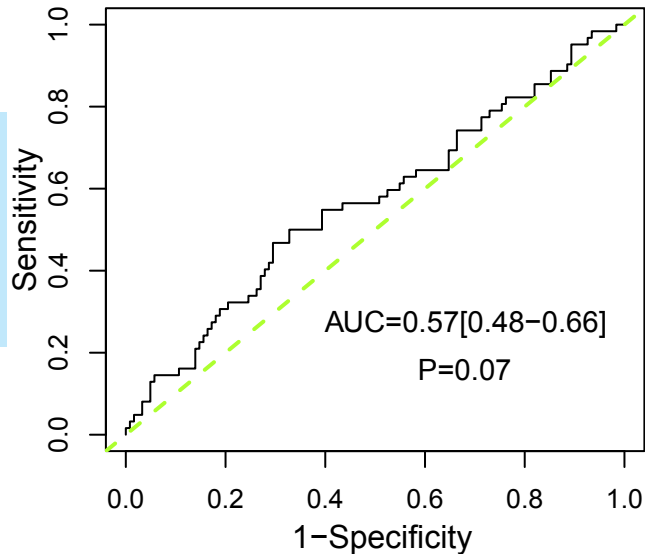

(B)

BC Diagnosis (more than 2 years)

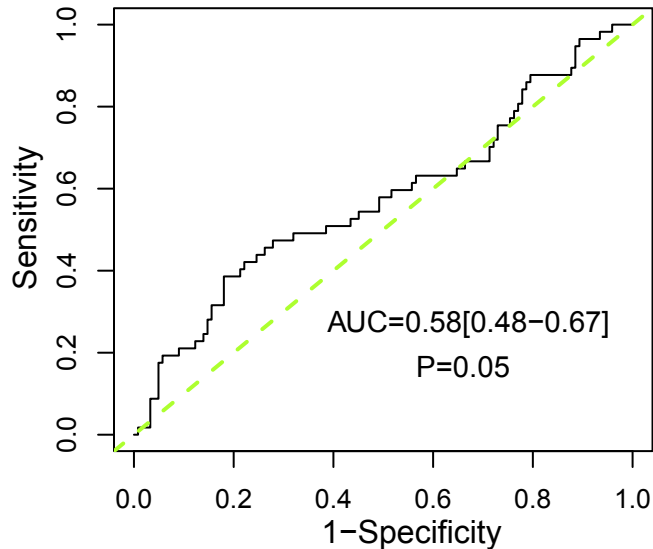

Supplement: Additional file 12 — ROC curve of the identified signature on the UKCTOCS dataset, separated around the diagnosis time of less than/greater than 2 years. [file gm567-S12.pdf]

(A)

BC (Incidence)

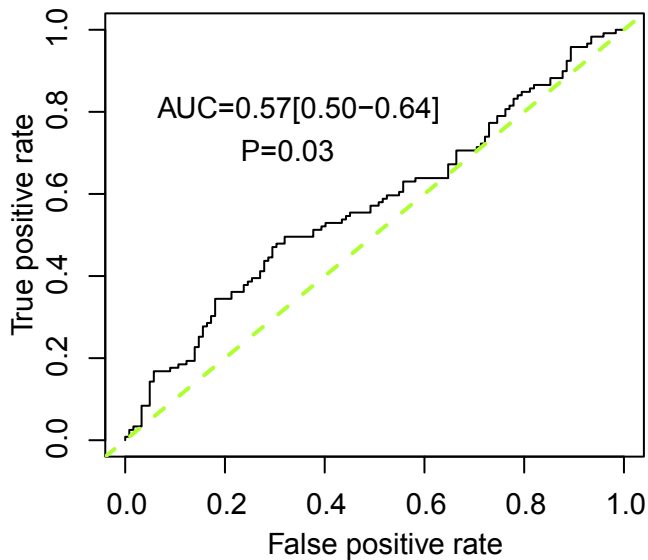

(B)

BC (Death)

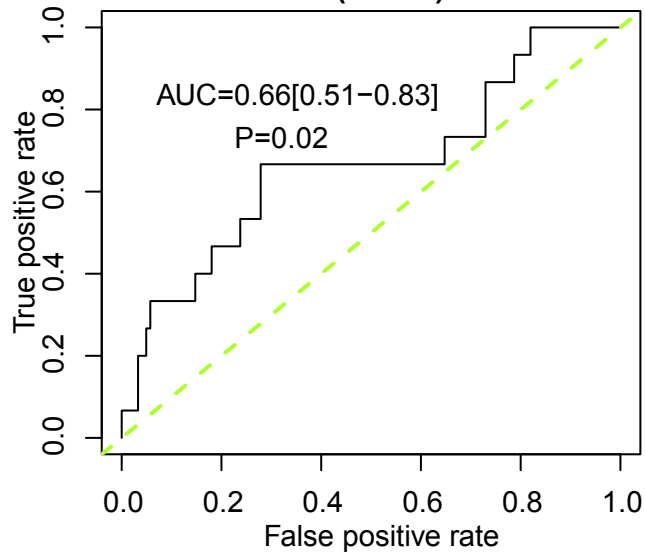

Supplement: Additional file 17 — ROC curve of the identified signature on the UKCTOCS dataset samples, without a family history of breast cancer, separated into breast cancer incidence and breast cancer mortality. [file gm567-S17.pdf]

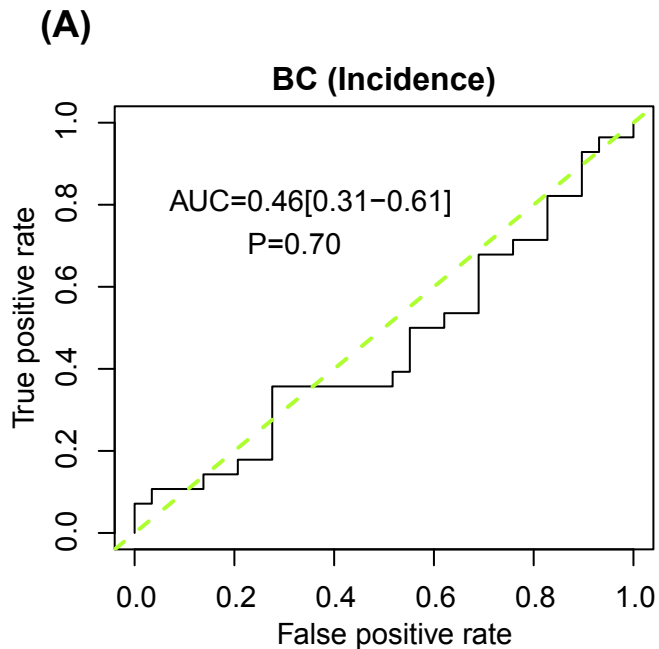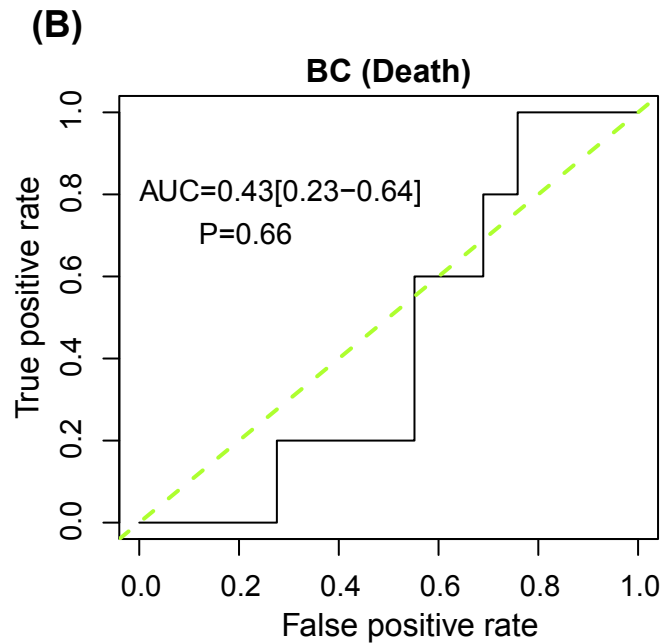

Supplement: Additional file 18 — ROC curve of the identified signature on the UKCTOCS dataset samples, with a family history of breast cancer, separated into breast cancer incidence and breast cancer mortality. [file gm567-S18.pdf]
